# Characterizing Cardiac Involvement in Chronic Kidney Disease Using CMR—a Systematic Review

**DOI:** 10.1007/s12410-018-9441-9

**Published:** 2018-01-31

**Authors:** Kenneth Mangion, Kirsty McDowell, Patrick B. Mark, Elaine Rutherford

**Affiliations:** 1 Institute of Cardiovascular and Medical Sciences, BHF Cardiovascular Research Centre, University of Glasgow, BHF Building, 126 University Place, Glasgow, G12 8TA UK; 2 Glasgow Renal & Transplant Unit, NHS Greater Glasgow & Clyde, Queen Elizabeth University Hospital, Glasgow, UK

**Keywords:** Cardiac magnetic resonance, Chronic kidney disease, T1 mapping, Strain

## Abstract

**Purpose of Review:**

The aim of the review was to identify and describe recent advances (over the last 3 years) in cardiac magnetic resonance (CMR) imaging in patients with chronic kidney disease (CKD). We conducted a literature review in line with current guidelines.

**Recent Findings:**

The authors identified 22 studies. Patients with CKD had left ventricular global and regional dysfunction and adverse remodeling. Stress testing with CMR revealed a reduced stress-response in CKD patients. Native T1 relaxation times (as a surrogate markers of fibrosis) are elevated in CKD patients, proportional to disease duration. Patients with CKD have reduced strain magnitudes and reduced aortic distensibility.

**Summary:**

CMR has diagnostic utility to identify and characterize cardiac involvement in this patient group. A number of papers have described novel findings over the last 3 years, suggesting that CMR has potential to become more widely used in studies in this patient group.

## Introduction

Chronic kidney disease (CKD) covers a wide spectrum of pathology, from early, subclinical changes in renal function in patients with multiple co-morbidities to end-stage renal disease (ESRD) where renal replacement therapy is required to sustain life [1]. In high income countries, the prevalence of CKD is approximately 10–13% [2] and estimates suggest that worldwide, in 2013, almost 1 billion people died as a result of CKD [3]. However, individuals with early CKD are more likely to die of cardiovascular (CV) disease than they are to progress to ESRD [4]. Increased CV risk is due to a combination of both traditional risk factors (e.g., hypertension, diabetes, coronary artery disease) and novel factors (e.g., subclinical ischemia, arteriosclerosis, arterial stiffening, hemodynamic insults) [3–5]. As CKD progresses, the risk of CV disease becomes increasingly exaggerated—with an increasing excess of arrhythmia, sudden cardiac death and congestive cardiac failure [5–7].

This excess of CV disease is intrinsically linked to cardiac structural and functional abnormalities, which start to develop early in CKD. These include left ventricular hypertrophy (LVH), ventricular dilation, cardiac dysfunction, and myocardial fibrosis, which together are sometimes referred to as a “uremic cardiomyopathy” [6, 7]. Detection and ultimately reversal of these cardiac abnormalities is an important goal for improving the morbidity and mortality of CKD patients. In recent years, the use of cardiac magnetic resonance (CMR) imaging to detect these abnormalities has been an area of development and CMR use as an investigative tool in this patient group has gained traction.

**Fig. 1:**
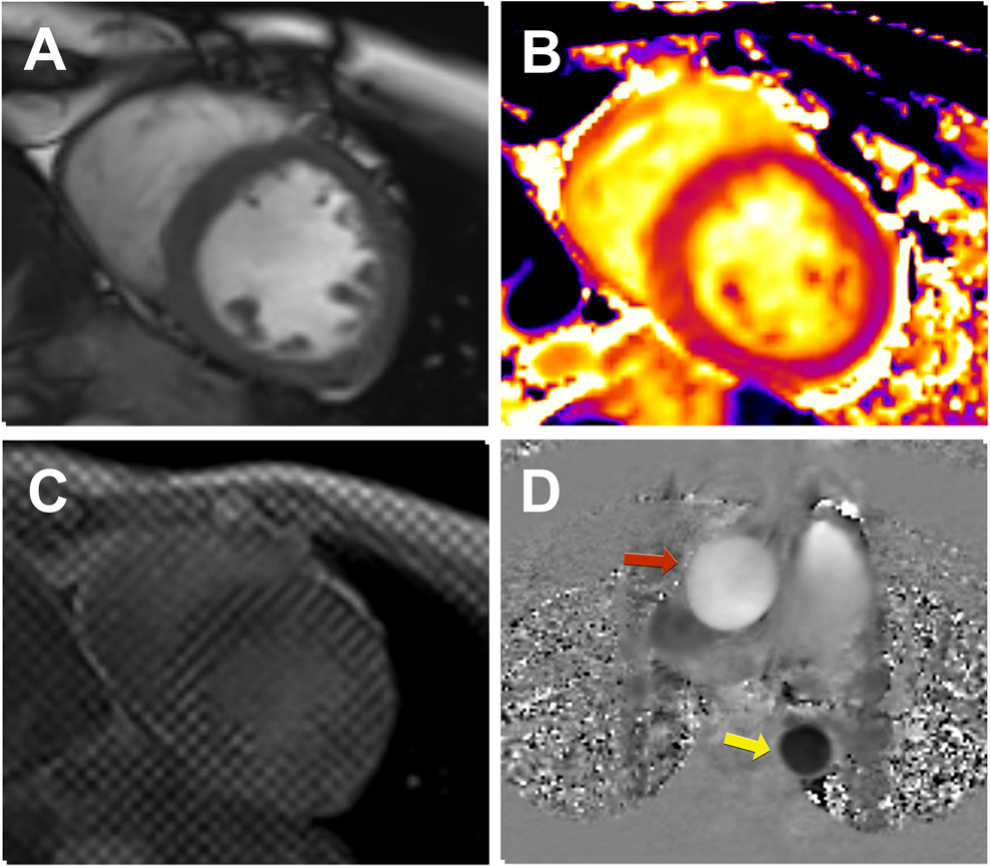
Utility of CMR in patients with chronic kidney disease. A multiparametric (contrast-free) scan of a participant of the Cardiac Uraemic fibrosis Detection in DiaLysis patiEnts study (CUDDLE study ISRCTN99591655) demonstrates the utility of CMR to assess various indices of cardiovascular function. **a**–**c** Mid-left ventricular short axis slices. **a** Cine imaging (balanced-steady state free precession) used to assess cardiac volumes, mass, and function. **b** T1 parametric mapping (in this case a Modified Look-Locker Inversion Recovery, (MOLLI) sequence) is a technique typically acquired in mid-diastole with the potential to identify diffuse myocardial fibrosis. **c** Tagged CMR is considered to be the gold standard for myocardial strain through post-processing with harmonic phase algorithm. **d** Phase-contrast imaging is utilized to investigate aortic distensibility (red arrow–ascending aorta, yellow arrow–descending aorta)

CMR imaging provides multiparametric information in a single scan, uniquely integrating function with pathology. CMR is the gold standard for quantification of myocardial volumes and function [8]. CMR has superior accuracy and precision when compared with echocardiography [9, 10]. In the CKD population, CMR also has lower inter-observer variability than echocardiography and is thus ideally suited to clinical research [11]. An example of a typical CMR research exam in this patient group is depicted in Fig. 1. In our group, we aim to keep CMR scans under 45 min, reaching a compromise between patient comfort and imaging yield.

There are a number of techniques available for characterizing myocardial function (volumes, ejection fraction (EF), peak systolic strain, strain rate) and pathology (contrast-enhanced CMR, parametric mapping) (Table 1).

**Table 1:** CMR techniques to assess myocardial features of CKD

CMR sequence	Assessment	Utility	Comments
Cine imaging	LV mass	Reference standard	No geometrical assumptions with CMR.
	LV function, RV function	Reference standard	Volumes, EF are dependent on heart rate, bloodpressure, inotropic state.
	Atrial size		No geometrical assumptions if short axis stack used.
	Cine-strain	Strain is theoretically more tightly linked with pump-function than LVEF.	Cine-strain segmental analysis is not accurate enough for clinical use.
Contrast-enhanced MR	Late gadolinium enhancement (scar/fibrosis)	Standard in clinical CMR practice to identify focal or diffuse scar	Gadolinium-based contrast agents contraindicated in patients with eGFR < 30 ml/min/1.73 m^2^
T1 mapping (pre- and post- contrast)	Diffuse fibrosis, chronic scar, inflammation	Longitudinal relaxation (T1, ms). Pre and post gadolinium contrast T1 mapping and hematocrit can calculate segmental Extra-cellular volume fraction. Can be used to identify diffuse fibrosis, edema.	Gadolinium-based contrast agents contraindicated in patients with eGFR < 30 ml/min/1.73 m^2^
T2 mapping	Oedema	Transverse decay (T2, ms). Standard in clinical CMR or identifying myocardial edema.	
T2* mapping	Iron overload	Gold standard in non-invasive assessment of iron overload.	Can only be reliably used at 1.5T. Higher artifact rate at 3.0T.
Bespoke strain techniques (tagging, phase-contrast imaging)	Peak systolic strain, strain rate, early diastolic strain rate	Tagging is the gold standard for strain assessment.	Time consuming analysis. Bespoke strain techniques prolong imaging time.
Phase-contrast imaging	Aortic pulse wave velocity, flow	Standard CMR assessment of aortic flow.	
Adenosine-stress perfusion imaging	Inducible perfusion defect	Typically requires clinician supervision	Gadolinium-based contrast agents contraindicated in patients with eGFR < 30 ml/min/1.73 m^2^
Dobutamine stress imaging	Inducible wall-motion abnormality	Typically requires clinician supervision	Theoretical risk of inducing ventricular arrhythmias, angina.
Blood oxygen level dependent imaging	T2* signal reduction with reduction in myocardial oxygenation	Non- invasive assessment of myocardial microcirculation	Still a research tool, not routinely available in clinical practice.

*LV* left ventricle, *EF* ejection fraction, *eGFR* electronic glomerular filtration rate

CMR has a number of important limitations, including longer examination times than with echocardiography or computed tomography, and lower temporal resolution than echocardiography. CMR is unsuitable for claustrophobic patients, or for patients with hemodynamic instability, as the patient is removed from direct care. Patients with intra-cranial and intra ocular ferromagnetic objects, as well as cochlear implants and certain cardiac pacemakers are contraindicated from undertaking CMR examination [12]. However, despite these limitations, CMR remains an attractive tool for characterizing cardiac pathology within the CKD population and this article aims to summarize recent developments within the field as well as providing a synopsis of recent studies in this population that utilized CMR.

### Methodology

**Fig. 2:**
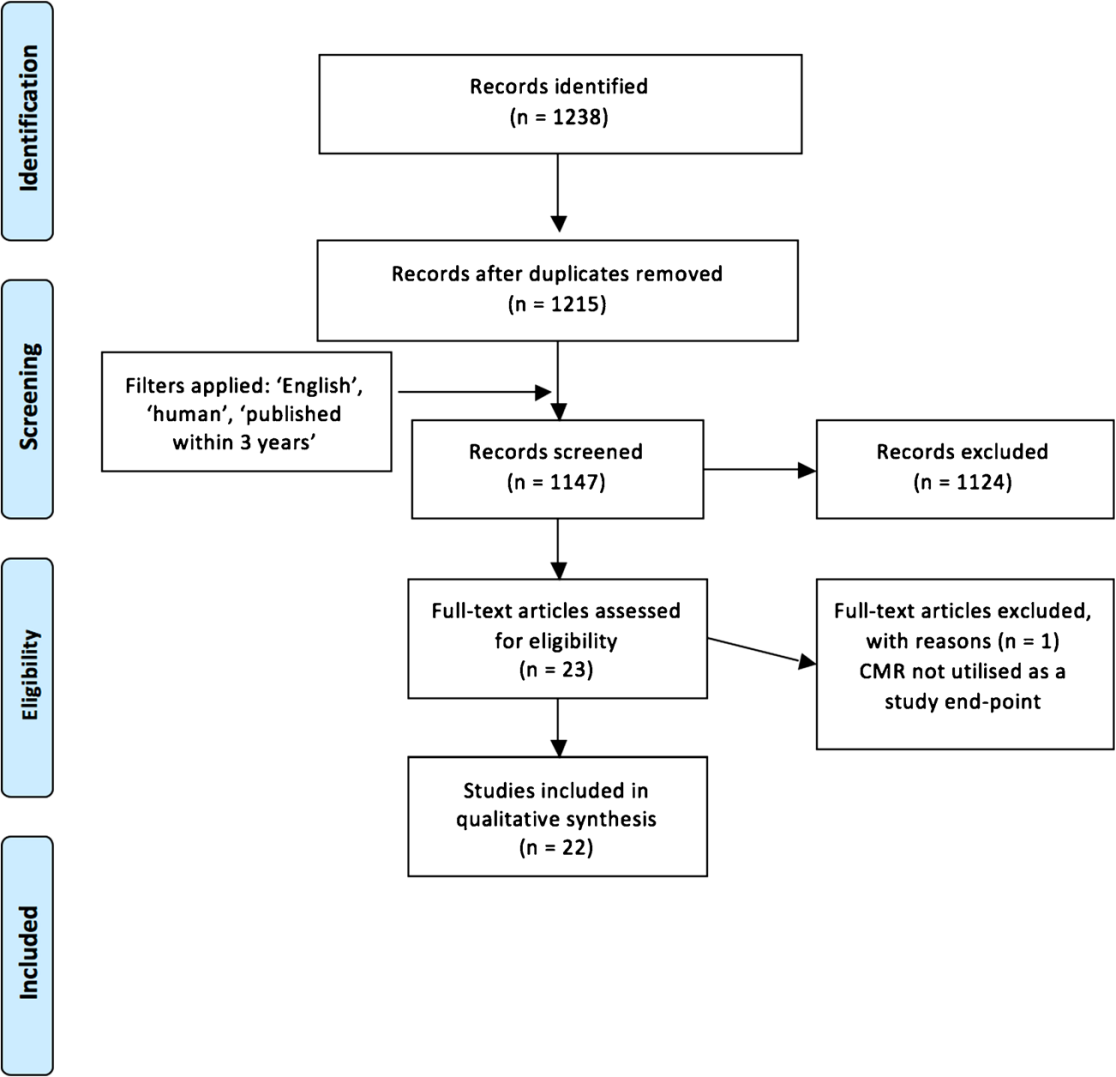
Flow chart of review process

In order to provide a focused update on the developments and findings from CMR studies characterizing cardiac involvement in CKD over the last 3 years, a systematic literature review was performed. This was done in accordance with the PRISMA [13] guidelines by two researchers (KM, KMcD) (Fig. 2) who independently searched PubMed and Web-of-Science using the following keywords and variations on them: ‘Renal failure’, ‘Chronic kidney disease’, ‘Hemodialysis’, ‘Renal’, ‘Renal transplant’, ‘Cardiac magnetic resonance’, ‘cardiac imaging’.

Our search was restricted to peer-reviewed journals and human subjects. Editorials, reviews, studies with fewer than 10 patients or those not published in English were excluded.

### Study Selection

Abstracts of all potential titles were reviewed by KM and KMcD. References of relevant reviews and all full papers were searched to retrieve any additional papers, repeating the process until no new papers were found (Table 2).

**Table 2:** Relevant articles published in the last 3 years assessing cardiac involvement in CKD utilizing CMR

Author	Year	Population	Renal patients	Main findings
Incidental findings				
Rutherford et al., [14]	2017	ESRD	161	15% clinical significant incidental findings in this population.
Myocardial structure and function				
Arnold et al., [15]	2016	ESRD (pediatric)	25	Compared to controls, pediatric ESRD patients had higher LV mass, reduced cardiac output.
Buchanan et al., [16••]	2016	ESRD	12	Intra-dialytic CMR revealed transient segmental LV systolic dysfunction.
Dundon et al., [17]	2014	Post-renal transplant	18	AV fistula ligation post-transplant was associated with a regression in LV mass, improvement in RV function.
Friesen et al., [18]	2015	ESRD	11	Nocturnal hemodialysis was associated with regression in LV and RV mass.
Odudu et al., [19]	2015	ESRD	73	Patients undergoing cooler HD experienced a regression in LV mass and had improved aortic distensibility.
Odudu et al., [20]	2016	ESRD	54	ESRD patients had reduced magnitudes of peak systolic strain as assessed using tagged CMR, reduced aortic distensibility, and higher LV mass, when compared to controls.
Patel et al., [21]	2014	Renal transplant	119	Left ventricular hypertrophy and left atrial dilatation pre-transplant were independent predictors of mortality
Ross et al., [22]	2016	ESRD	67	LV remodeling at 1 year might be related to volume and pressure overload related to hemodialysis.
Sarak et al., [23]	2017	ESRD	57	Change in mean arterial pressure correlated with change in indexed LV mass over a 1 year period of either conventional or nocturnal hemodialysis.
Wald et al., [24]	2014	ESRD	56	Ventricular dilatation appears to be an independent determinant of LV mass
Wald et al., [25]	2016	ESRD	67	Patients switched to nocturnal HD experienced a regression in LV mass when compared with patients on conventional HD.
Ischemia assessment				
Parnham et al., [26]	2015	Renal transplants	20	Myocardial perfusion reserve index was reduced in renal transplant recipients when compared with hypertensive controls using adenosine-stress CMR.
Parnham et al., [27]	2016	ESRD, Renal transplant	23, 10	CKD patients have a reduced myocardial oxygen response to adenosine stress, potentially due to renal function
Ripley et al., [28]	2014	ESRD	41	Dobutamine stress CMR is well tolerated and safe in patients with ESRD with no serious adverse effects.
Advanced CMR assessment				
Edwards et al., [29•]	2015	CKD	43	Patients with early CKD had higher T1 and ECV values, and lower global longitudinal strain when compared with hypertensive patients and healthy controls.
Gimpel et al., [30]	2017	ESRD	20	Phase-contrast CMR identified diastolic dysfunction
Graham-Brown et al., [31•]	2016	ESRD	35	ESRD on long-term dialysis had higher T1 relaxation times and reduced peak longitudinal and circumferential strain when compared with healthy volunteers.
Graham-Brown et al., [32]	2017	ESRD	20	T1 is unaffected by patient fluid status; T1 analysis is a reproducible technique, accounting for intra- and inter- observer variability, and inter-center variability.
Holman et al., [33]	2017	ESRD	10	T2* CMR identified hepatic but not cardiac iron loading in 80% of patients taking iron supplementation.
Rutherford et al., [34•]	2016	ESRD	33	ESRD patients had higher T1 relaxation times and reduced peak longitudinal strain when compared with healthy volunteers.
Tolouian et al., [35]	2016	ESRD	17	T2* CMR identified hepatic but not cardiac iron loading in 50% of patients taking iron supplementation.

*LV* left ventricle, *ESRD* end stage renal disease, *CKD* chronic kidney disease, *CMR* cardiac magnetic resonance

## The Utility of CMR to Characterize Myocardial Involvement in Patients with Chronic Kidney Disease

### Myocardial Remodeling

Utilizing CMR, pediatric and adult patients with CKD and incident HD have recently been reported to have LVH, when matched to healthy controls [15, 20]. In fact, it is well known that a significant proportion of patients with CKD have LV hypertrophy, which is associated with increased mortality [36, 37] and cardiac arrhythmia [36]. This has led to LV mass regression being the focus of a number of clinical trials [19, 38], as, theoretically, this should be associated with a reduction in mortality and sudden cardiac death. Surprisingly, a recent meta-analysis [39] looking at 73 trials and over 6500 patients did not identify a clear association between LV mass regression and mortality. A possible explanation for this is that most of the studies (87%) utilized echocardiography to quantify LV mass. One of the advantages of CMR is the use of a retrospectively ECG-gated axial stack of cine imaging to accurately delineate endo- and epicardial borders at end-diastole and systole [40]. This obviates the need for assumptions that the left ventricle is an ellipsoid object, such as with the bi-plane equation used in echocardiography. In the future, a meta-analysis of only CMR studies considering LV mass regression would of course be of interest, but currently there are insufficient numbers of CMR studies to make this a meaningful exercise.

Avoidance of fluid overload and targeting blood pressure control in patients on HD may prevent LV dilation and remodeling, and result in LV mass regression—a recent observational study demonstrated that a greater indexed LV mass was independently associated with greater systolic blood pressure and with greater LV indexed end diastolic volume [24].

As an alternative to conventional hemodialysis, nocturnal hemodialysis holds promise. Two recent studies have investigated whether the avoidance of significant fluid shifts by performing dialysis overnight for longer periods of time or more frequently than three times per week might result in myocardial remodeling and regression in LV hypertrophy. Both studies demonstrated LV mass regression [18, 25], this may have been related to better BP control [23]. When the authors of one of these studies [25] looked at associates of adverse cardiac remodeling in their study population over the year of their study follow up they found volume and pressure overload were the biggest factors contributing to negative myocardial changes. Following this logic, it is not surprising that more frequent, or longer nocturnal dialysis led to LV mass regression [22]. Neither of these two studies was fully randomized but their findings were in keeping with an earlier randomized study [41]. While these findings are encouraging, the next step would be to establish whether this CMR detected myocardial remodeling translates into improved clinical outcomes.

Further insights into the acute effects of HD and hemodiafiltration on myocardial function were recently described in an ambitious study where patients underwent CMR imaging during their dialysis therapy [16••]. The authors of this study must be commended for overcoming multiple logistical challenges in order to address this issue. Both modalities resulted in reduced global and regional myocardial contractile function during treatment, with the severity being proportional to ultrafiltration rate and BP reduction with partial recovery post therapy. Performing CMR during dialysis is no simple feat and must be relatively arduous for both patients and professionals involved. For this reason, it is highly likely that studies of this nature will involve very small numbers of patients only. A large-scale clinical trial involving this method of imaging is currently not practical.

However, slightly larger studies are possible in the dialysis population by making small changes to in-center dialysis regimens. For example, Odudu has demonstrated in a study of 73 incident HD patients that cooling dialysate fluid could potentially slow cardiac remodeling, with patients randomized to cooled dialysis versus normal HD regimen experiencing a reduction in LV mass and volumes at 1 year [19]. In this study, there was no improvement in ejection fraction which was the study primary outcome, however peak systolic strain, which is perhaps a more sensitive marker of cardiac function, was preserved by the intervention. Assessment of myocardial strain using CMR is a developing area of interest within the renal population and is discussed further later in this review.

Candidates for renal transplantation could potentially be risk-stratified based on CMR imaging data recently reported [21], with pre-transplantation LV hypertrophy and left atrial dilatation being independent predictors of mortality post-transplantation. A possible treatment for LV hypertrophy regression post-transplantation is the ligation of any patent arterio-venous fistulae [17].

### Myocardial Tissue Characterization

One of the big challenges of imaging the renal population is that it is not possible to use gadolinium-based contrast agents in ESRD patients because of concerns regarding nephrogenic systemic fibrosis [42]. This has therefore stimulated interest in the nephrology community in alternative techniques to quantify tissue abnormalities such as parametric mapping. Native T1 mapping measures the longitudinal relaxation of hydrogen ions after applying inversion magnetization pulses [43, 44]. If renal function is good enough to permit the use of gadolinium-based contrast agents, post-contrast T1 maps may be acquired, and utilizing the serum hematocrit, then the extra-cellular volume fraction can be calculated [45]. If native T1 time is prolonged, then this may represent a tissue abnormality such as an increase in interstitial space (fibrosis, amyloid deposition), or edema (myocardial infarction or inflammation, e.g., myocarditis). T2 mapping measures the transverse decay time of hydrogen ions, and the main cause of raised T2 values is edema (myocardial infarction or inflammation) [46]. T2* mapping has been developed in view of the paramagnetic properties of iron (present as ferritin and hemosiderin) in the myocardium and liver [46]. Thus, iron deposition (from iron supplementation, or due to intra-myocardial hemorrhage) results in a low T2* signal on parametric mapping.

CKD is associated with myocardial interstitial fibrosis [47]. Recent research has described how native T1 values were higher than controls in patients with early stage CKD [29•], ESRD on hemodialysis for < 6 months [34•] and in patients on hemodialysis for > 12 months [31•]. In fact, looking at septal native T1 values (ms) across the three publications, native T1 values increase with a longer duration of hemodialysis supporting the fact that the degree of interstitial fibrosis is proportional to the disease duration.

A further paper recently published describes native T1 mapping as a robust technique with excellent intra-observer, inter-observer, and inter-study variability assessment [32]. T1 acquisition on T1MES phantoms [48] between two centers, as well as inter-center analysis of native T1 datasets acquired in patients with CKD-derived comparable T1 values, supporting the use of parametric mapping as an exploratory endpoint in multicenter studies. However, tissue correlation of native T1 mapping in the renal population has not yet been done and some may argue that before it becomes a standard imaging sequence in renal CMR studies this hurdle should be overcome.

T2* mapping has been applied to patients with CKD to investigate for potential iron overload. This is as these patients get erythropoietin and parenteral iron infusions for iron deficiency to due reduced iron absorption and release from tissues [49]. Interestingly, 50% of the patients in one group [35] and 80% in another recently described group [33] had reduced hepatic T2* signal in keeping with iron overload. There was, however, no evidence of myocardial iron loading in either study.

### Myocardial Strain Assessment

There are several techniques for assessing myocardial strain with CMR. This is of importance in the renal population, as there is emerging evidence that patients with renal disease have abnormal strain despite normal LV ejection fraction. The bespoke strain methods include phase-contrast [50], tagging [51], displacement encoding with stimulated echoes (DENSE) [52, 53], and cine-derived strain [54, 55].

Myocardial tagging derives strain estimation by imaging and tracking tissue markers (“tags”) induced by changes to the tissue magnetization [51]. Tagging is considered as the gold standard reference method for CMR strain [56]. Feature-tracking (FT) involves retrospective motion tracking of cine imaging. While vendor dependent, most cine-strain techniques derive strain by tracking the displacement of the endo- and epicardial borders, while taking into account columns of pixels in the myocardium [54]. While the derivation of strain from cine imaging has utility in keeping scanning time short in this patient group, there is a trade-off with greater measurement variability when compared to bespoke strain techniques [55, 57].

Patients with early CKD have been described to have reduced global longitudinal strain and strain rate compared with controls utilizing FT [29•]. This trend was observed in HD patients < 6 months vintage utilizing tagging [20] and FT [34•], and in HD patients > 6 months vintage [31•]. Interesting insights from a pediatric population [30] utilizing phase-contrast describe markedly reduced early diastolic ventricular function in CKD patients without LV hypertrophy on CMR. Abnormalities in myocardial strain could therefore represent some of the first detectable changes in the hearts of CKD patients and in future may even have a role in screening or identifying patients with subclinical disease, who are theoretically at greater risk of more advanced cardiac disease.

### Assessment of Myocardial Ischemia

Myocardial ischemia assessment with CMR can be dichotomized into the assessment of inducible wall-motion abnormalities by infusing dobutamine or by the use of vasodilator-stress perfusion testing to identify an inducible perfusion defect. The use of dobutamine infusion results in a rise in the myocardial metabolic demand, through chronotropic stimulation. Myocardial segments subtended by a stenosed coronary artery are unable to meet the metabolic demands, and appear hypokinetic [58]. Dynamic LV outflow tract obstruction can occur, resulting in false positive wall motion “abnormalities.”

Vasodilator-stress testing makes use of vasodilator agents such as adenosine or regadenoson causing a 3-5× fold coronary vasodilatation in normal coronary arteries, while stenosed vessels are unable to vasodilate to the same extent. The infusion of gadolinium contrast at peak stress and at rest enables the clinician to identify regions of inducible ischemia. This is one of the most sensitive non-invasive techniques available for the identification of myocardial ischemia [59, 60]. This technique is however unsuitable in patients with reduced eGFR [58]. Blood oxygen level dependent (BOLD) CMR is similar to T2* mapping in making use of the paramagnetic effect of deoxyhemoglobin to observe a signal drop, in tissues with reduced tissue oxygenation, due to epicardial coronary artery stenosis or impaired microcirculation [61].

The safety and feasibility of dobutamine stress CMR was recently described in patients with ESRD [28]. 93% of the patients achieved 85% of the age-predicted heart rate. Ten percent of the completed stress scans were positive for inducible wall motion abnormalities and there were no serious adverse events reported. Adenosine-stress CMR was utilized in renal transplant recipients with no known coronary artery disease, with the myocardial perfusion reserve index (an assessment of perfusion) being reported as being lower in transplant recipients when compared with hypertensive controls [26]. CMR angiography was also acquired in this patient group, with 35% of the patients having 50% coronary artery stenosis. Adenosine-stress CMR coupled with BOLD was investigated in patients with ESRD and in transplanted patients without known coronary artery disease [27]. The mean signal intensity in both patients with CKD and in patients post-transplant were lower than that acquired in hypertensive individuals or in normal controls, with the lowest myocardial oxygen response to stress being in CKD patients on dialysis, then in CKD patients pre dialysis. Renal transplantation recipients appeared to have an improved tissue oxygen response, but still significantly different from healthy volunteers.

### Aortic Stiffness

Arterial stiffening, through arteriosclerosis, is one of the earliest signs of subclinical cardiac involvement of CKD [62]. CMR enables the measurement of aortic pulse wave velocity by contouring the ascending and descending aorta on phase-contrast MR to derive the temporal shift and thus pulse wave velocity [63]. Aortic distensibility can be calculated by contouring a short axis view of the ascending aorta on cine imaging and obtaining an aortic pulse pressure via brachial cuff [63].

Patients with a 3 months HD vintage had reduced aortic distensibility and increased aortic pulse wave velocity when compared to healthy controls [20]. Aortic distensibility was preserved in a study group of HD patients using cooled dialysate fluid [19]. Studies suggest that increased arterial stiffness and reduced aortic distensibility are pathophysiologically associated with arterial hypertension and an increase in LV loading. This in turn leads to LV hypertrophy, which is in turn associated with adverse events. Thus, detecting reduced aortic distensibility in patients with CKD has the potential to be an early biomarker of circulatory dysfunction.

### Non-Cardiac Findings on a Cardiac Scan

While the main focus of CMR scans is to assess myocardial changes in CKD patients, the initial coronal, longitudinal, and transverse planes of the thoracic cavity and upper abdomen are acquired by radiographers for study planning. This leads to imaging a significant volume of the patients’ bodies beyond the organ of interest, which may reveal important pathology. While this is not directly relevant to characterizing myocardial disease involvement, we felt this is an important point to discuss in view of increased use of CMR in studies in patients with CKD. Recently, a paper reported the incidence of non-cardiac findings in a CKD stage 5 population as being seven times greater than what was previously reported in an all-comers CMR study [14, 64]. While this study was limited by its retrospective nature, researchers utilizing CMR in renal populations should consider its findings seriously. It is the responsibility of researchers to ensure that there are robust reporting pathways in place for any obtained images and that all individuals are fully informed about the potential consequences of incidental findings.

### Limitations and Challenges of CMR in this Population

While there has been excellent progress in characterizing cardiac involvement in chronic kidney disease over the last 3 years, there are a number of important limitations, which the authors encountered on reviewing these papers. Most of the studies carried out were observational in nature, not randomized, and investigated small numbers of patients (*n* < 25). There is a lack of prognostic utility of these novel imaging markers described in the review. There is potential through collaborative work of randomized, multicenter studies assessing the prognostic utility of these markers on composite endpoints.

Another important challenge in this patient group is the relation of timing of imaging to dialysis and the resultant fluctuant fluid changes, which could give differing hemodynamic measurements depending on loading conditions. This is especially important in multicenter studies.

## Conclusions

Over the last 3 years, there have been a number of studies investigating the CV system in patients with CKD utilizing CMR. This shows that CMR is a multiparametric tool with diagnostic utility in this patient group. While further work needs to be undertaken in these patients, there is potential for CMR-derived endpoints to be utilized in larger studies in this group of patients.

## Abbreviations

CV: Cardiovascular
CKD: Chronic kidney disease
CMR: Cardiac magnetic resonance
EF: Ejection fraction
ESRD: End stage renal disease
LV: Left ventricle
LVH: Left ventricular hypertrophy
